# Ganaxolone: A New Treatment for Neonatal Seizures

**DOI:** 10.3389/fncel.2017.00246

**Published:** 2017-08-22

**Authors:** Tamara Yawno, Suzie L. Miller, Laura Bennet, Flora Wong, Jonathan J. Hirst, Michael Fahey, David W. Walker

**Affiliations:** ^1^Ritchie Centre, Hudson Institute of Medical Research Clayton, VIC, Australia; ^2^Department of Obstetrics and Gynaecology, Monash University Clayton, VIC, Australia; ^3^Department of Physiology, The University of Auckland Auckland, New Zealand; ^4^Department of Paediatrics, Monash University Clayton, VIC, Australia; ^5^School of Biomedical Sciences and Pharmacy, University of Newcastle Callaghan, NSW, Australia; ^6^School of Health and Biomedical Sciences, RMIT University Bundoora, VIC, Australia

**Keywords:** neurosteroids, ganaxolone, phenobarbitone, neonatal seizures, hypoxic-ischemic encephalopathy, neuroprotection

## Abstract

Neonatal seizures are amongst the most common neurologic conditions managed by a neonatal care service. Seizures can exacerbate existing brain injury, induce “de novo” injury, and are associated with neurodevelopmental disabilities in post-neonatal life. In this mini-review, we present evidence in support of the use of ganaxolone, a GABA^A^ agonist neurosteroid, as a novel neonatal therapy. We discuss evidence that ganaxolone can provide both seizure control and neuroprotection with a high safety profile when administered early following birth-related hypoxia, and show evidence that it is likely to prevent or reduce the incidence of the enduring disabilities associated with preterm birth, cerebral palsy, and epilepsy. We suggest that ganaxolone is an ideal anti-seizure treatment because it can be safely used prospectively, with minimal or no adverse effects on the neonatal brain.

## Neonatal seizures

Seizures in neonates are relatively common; in fact, this is the stage of life they are most likely to occur (Khanna et al., 2013). Their presence is often the first sign of significant brain dysfunction. Although often of short duration, they are nevertheless powerful predictors of long-term cognitive and developmental impairment (Glass, 2014). Neonatal seizures are associated with cognitive, intellectual, behavioral, and sensorimotor deficits (Glass, 2014) independent of any other associated brain damage. Thus, there is a great need to find an effective anti-seizure therapy.

Identification of seizure in neonates is often hard, and treatment decisions remain problematic. It is uncertain whether it is better to treat electrographic or clinical seizures associated with cerebral hypoxia-ischaemia (Bjorkman et al., 2010). Despite more than 60% of neonatal seizures being solely electrographic, without obvious physical manifestations, most management focuses on clinical seizures (Bye and Flanagan, 1995). However, the increased use of brain activity monitoring through a continuous recording of the electroencephalogram (EEG) has revealed that the actual rate of neonatal seizures is at least double the number reported by clinical observation alone (Glass, 2014). With the increasing use of bedside monitors to continuously record EEG in neonatal intensive care units, we have transitioned from a reliance on clinical seizure events (fits/jerks/abnormal body movements) to a more comprehensive approach that includes EEG identification of electrographic seizures and subtle/subclinical seizures (Glass, 2014).

Electrographic seizures are highly correlated with neurological compromise (McBride et al., 2000). Almost half of term infants with seizures are at risk of developing cerebral palsy, cognitive deficits, and epilepsy (Glass and Wirrell, 2009). In human infants, even a brief period of seizures is associated with altered brain development, long-term structural changes, increased seizure susceptibility, and impaired cognition (Holmes, 2009). While the neurological outcome is highly influenced by the underlying etiology of seizures, there is substantial evidence from human studies, and animal models that seizures independently induce, or worsen, brain injury (Bjorkman et al., 2010; Srinivasakumar et al., 2015). In the developing brain, seizures may increase neuronal injury by increasing metabolic demand, altering cerebral oxygenation, inducing a further release of excitatory neurotransmitters, and altering neuronal connections (Wasterlain et al., 2013).

Nonetheless, current treatment of neonatal seizures, only reduces clinical seizure events in a subset of infants, while the underlying electrographic discharges often remain unchanged (Glass, 2014). EEG assessment of neonatal seizures is considered gold-standard, and anti-seizure treatments must target a reduction in electrographic seizures. The clinical evidence is clear that reduction of the incidence and duration of electrographic seizures in neonates also reduces the severity of brain injury on MRI, and improves neurofunctional outcomes (Srinivasakumar et al., 2015).

## Current treatment of neonatal seizures

Phenobarbitone was discovered as an anti-seizure medication for adults in 1912 and was progressively adopted into clinical practice for neonates, without examination of its neurodevelopmental effects. Phenobarbitone remains the first-line treatment for infants with seizures today (WHO) (World Health Organisation, 2011). The standard second-line treatment for neonatal seizures is phenytoin, another older generation drug. Phenobarbitone and phenytoin do not provide good electrographic seizure control, with >50% of neonates still demonstrating electrographic seizures despite medication (Painter et al., 1999; Boylan et al., 2002, 2004; Rennie and Boylan, 2007).

Each of these medications has documented side effects. Phenobarbitone may cause respiratory depression and impaired myocardial function, while phenytoin is known to cause cardiac arrhythmia and hypotension (Levene, 2002). Antenatal exposure to phenobarbitone or phenytoin is linked to reduced brain volume in infancy and subsequent learning deficits (Dessens et al., 2000). Animal studies show that phenobarbitone induces cell death in gray and white matter and reduces synaptic connectivity in the developing, immature brain (Bittigau et al., 2003; Dringen, 2005; Forcelli et al., 2012). In addition, phenobarbitone is associated with significant deficits in motor ability and cognition and can increase anxiety behaviors (Brodie and Kwan, 2012; Forcelli et al., 2012). A meta-analysis in human infants found that anticonvulsant therapy following perinatal asphyxia did not significantly reduce seizure burden, and did not improve outcomes (Evans et al., 2007). A recent Cochrane review (Young et al., 2016) revealed that there is low to very low-quality evidence addressing the use of barbiturate (phenobarbitone) for the treatment of seizures in late preterm/ term infants following perinatal asphyxia. The analysis found that there was no reduction in mortality and little data addressing the long-term consequences of barbiturate use in neonates, suggesting that future studies should address clinically important reductions in mortality and their long-term neurological impairments. Ongoing questions surround the clinical efficacy and safety of phenobarbitone for the treatment of neonatal seizures, which prompts the critical need for alternative therapeutic options.

### Neurosteroids

Neurosteroids derived from progesterone, such as allopregnanolone, are endogenously produced neurohormones that interact with GABA^A^ receptors and increase CNS inhibition (Belelli and Lambert, 2005; Reddy and Rogawski, 2012). The high levels of neurosteroids in the brain before birth are due to placental production of progesterone and other precursor steroids which are rapidly converted to pregnane steroids such as allopregnanolone in fetal brain until the time of birth (Nguyen et al., 2003). Allopregnanolone, also known as 5α-pregnan-3α-ol-20-one or 3α,5α-tetrahydroprogesterone (3α,5α-THP), as well as brexanolone (Belelli and Lambert, 2005; Kanes et al., 2017), positively modulates the GABA^A^ receptor causing a global inhibition of CNS activity. We have interrogated the neurosteroid synthetic pathway, to identify candidate agents for neuroprotection, specifically following hypoxia-ischemia. We have shown that in fetal life allopregnanolone not only promotes brain growth and protects against hypoxic damage, but it also provides a tonic suppression of brain activity, as revealed through EEG, and fetal motion and breathing movements (Nicol et al., 1999, 2001). There is a rapid loss of neurosteroid-induced inhibition of brain activity at birth after the placenta is removed, and neurosteroids such as allopregnanolone are cleared quickly from the neonate's circulation—the half-life of allopregnanolone is estimated to be 10 min (Johansson et al., 2002). The physiological sense of this rapid change of CNS neurochemistry is that the healthy term fetus “wakes up” once the tonic inhibition of *in utero* neurosteroids has is removed upon delivery. But for the post-hypoxic fetus, the loss of this physiological inhibition exposes the brain to oxidative stress and other neurochemical changes that increase excitability, and so, potentially, to the onset of seizures. For the prematurely born baby there is not only is this immediate loss of protective inhibition but a prolonged loss of the growth-promoting milieu that neurosteroids provide *in utero*.

The physiological importance of allopregnanolone for the brain immediately before birth is shown by the fact that inhibition of its synthesis is followed by greater spontaneous activity suggesting fetal arousal (Nicol et al., 2001), and by an increased incidence of isoelectric and spiking EEG activity following brief *in utero* asphyxia (Yawno et al., 2011). These effects are reduced by treatment with the synthetic pregnane analog alfaxalone (Yawno et al., 2011). Furthermore, the inhibition of allopregnanolone synthesis markedly increased asphyxia-induced cell death within the cerebellum and hippocampus (Yawno et al., 2007), injuries also diminished by administration of alfaxalone (Yawno et al., 2009).

Late gestation fetal hypoxia in ovine models (induced by occluding umbilical blood flow for 10 min) leads to electrographic seizures and increased cell death in the hippocampus, basal ganglia and cerebellum (Castillo-Melendez et al., 2004; Yawno et al., 2007), and severe brainstem injury (George et al., 2004). Prolonged seizures increase metabolic demand throughout the brain, increasing global, and local (seizure-associated) cerebral blood flow, and perhaps directing perfusion away from injured brain regions, and thus limiting the supply of glucose and oxygen necessary to sustain endogenous protective and repair mechanisms in damaged organs. Furthermore, prolonged seizures increase production of diffusible neurotoxic molecules (e.g., inflammatory cytokines, glutamate, lactate, glycerol, reactive oxygen species), which can lead to ischemic cell damage (Gunn and Bennet, 2009).

It is worth mentioning that the 21-hydroxylated by-product of allopregnanolone, tetrahydrodeoxycorticosterone (THDOC), is an endogenous inhibitory neurosteroid with similar actions to those of allopregnanolone (Reddy, 2010), and there are few synthetic analog of allopregnanolone that have therapeutic potential. Alfaxalone is an anticonvulsant and neuroprotective against excitotoxic brain injury (Mellon, 2007; Hirst et al., 2014), however, it is not the ideal neurosteroid replacement therapy for neonates because of respiratory depression and apnea associated with its use (Mellon, 2007). Allopregnanolone itself is also not ideal for at least two reasons. Firstly, it has a short half-life *in vivo*, and has little or no bioavailability when given orally. Secondly, it can undergo isoenzyme-driven back conversion to produce active intermediates such as dihydroprogesterone sulfate and pregnenolone sulfate, both of which are negative modulators of the GABA^A^ receptor, and thus, potentially, have pro-convulsant effects (Reddy, 2003).

## Ganaxolone as a novel neurosteroid-based anti-seizure drug

Ganaxolone is a synthetic 3β-methyl by-product of allopregnanolone (Bialer et al., 2010), which, like endogenous neurosteroids, modulates the activity of GABAergic interneurons via the benzodiazepine-binding site on GABA^A^ receptors. Unlike allopregnanolone, ganaxolone does not undergo back-conversion due to the 3β-methyl substituent in its chemical structure (Turkmen et al., 2011), thus avoiding the side effects, biotransformation and tolerance associated with allopregnanolone, and alfaxolone. In humans, ganaxolone can be given orally and sufficient blood levels can be maintained in human subjects with two- or three-times daily dosing (Monaghan et al., 1997). Furthermore, while allopregnanolone is readily oxidized at the 3α position resulting in accumulation of 3-keto metabolites that are mostly inactive at neuronal membrane receptor sites, by contrast, the methylation of ganaxolone at the 3β position (3α-hydroxy-3 β-methyl-5α-pregnan-20-one) prevents rapid metabolism and thereby provides for greater bioavailability (Lyden et al., 2000).

### Preclinical studies

Ganaxolone is the only neurosteroid evaluated so far for the treatment of epilepsy in humans (Nohria and Giller, 2007). It has shown to have neuroprotective properties in adult rodent seizure models. In chronically treated rats, ganaxolone tachyphylaxis does not develop (Reddy and Rogawski, 2000). A study in amygdala-kindled mice showed that suppression of behavioral and electrographic seizures was achieved with a single low dose of 6.6 mg/kg (Reddy and Rogawski, 2010). In mice, ganaxolone has also shown to be a more efficient anti-convulsant agent compared to two regular anticonvulsants, diazepam and valproate (Gasior et al., 2000). It effectively prevents the development of clonic seizures and development of sensitisation to the convulsive (tonic and clonic) and lethal effects of pentylenetetrazol; and chronic (over 5 days) pre-treatment was more efficacious than acute (one daily dose) (Gasior et al., 2000). Similarly, ganaxolone has a better anticonvulsant index than ethosuxamine, and valproate in generalized absence and partial seizures (Carter et al., 1997).

So far, there are only few studies that have examined the efficacy of ganaxolone on seizure management in the developing and immature brain. Liptáková and colleagues found that ganaxolone has antiepileptic effects against chemically induced seizures amongst postnatal rat pups, aged 9, 15, 30, and 60 days (Liptakova et al., 2000). Ganaxolone is also effective in reducing the development of infantile spasm at postnatal day 15 (Yum et al., 2014). Pharmacokinetic studies in mice, rats, rabbits, and dogs show that ganaxolone has a broad steady-state volume of distribution indicating that it distributes extensively into tissues, including the brain (Ram et al., 2001), and unlike other neurotoxic anticonvulsants, ganaxolone has shown to suppress seizures without compromising brain development and motor function in rats (Mares and Stehlikova, 2010). Pre- and postnatal developmental studies in mice, rats, and dogs show that ganaxolone does not alter fetal viability, or growth, and development from birth to weaning, and no teratogenic or genotoxic effects have been noted. Similarly, ganaxolone treatment (orally) to conscious dogs at a dose of 10 mg/kg did not alter their blood pressure or heart rate.

### Clinical safety and efficacy studies

Ganaxolone was given to a large cohort (>900) of subjects in doses up to 1,875 mg/day in adults and up to 54 mg/kg/day in children in Phase 1 and Phase 2 studies and clinical trials for epilepsy (Table 1). Single oral doses of 50–1,600 mg in healthy subjects resulted in plasma concentrations of 14–460 ng/ml (Bialer et al., 2013). Pediatric trials to date have focused on adults and children with refractory seizures (patients who continue to have seizures despite taking multiple anticonvulsant drugs). An open-label, add-on trial in 16 pediatric patients aged 6 months to 7 years with infantile spasms and continuing seizures found it was well tolerated with a good pharmacokinetic profile. Spasm frequency was reduced by at least 50% in 33% of these subjects, with an additional 33% experiencing some improvement, and one patient achieved seizure freedom (Kerrigan et al., 2000). A further open-label, non-randomized, dose-escalation add-on trial was conducted in 20 highly resistance pediatric and adolescent patients, aged 5–15 years. After 8 weeks of treatment, 47% had a substantial or moderate decrease in seizure frequency (Pieribone et al., 2007). Although these studies utilized small numbers of patients, they provide evidence that ganaxolone reduces seizures with acceptable tolerance, and safety profile even in those refractory to other therapy.

**Table 1:** Clinical trial in which Ganaxolone is used to treat epilepsy in children and adults.

	**Study title**	**Main objective**	**Country**	**Condition**	**Current status**	**Trial identifier**
**1**	A Two-year Open-label Extension Study of Ganaxolone in Patients With Drug-resistant Partial-onset Seizures	Two-year open-label extension study of ganaxolone as add-on therapy in adult patients with drug-resistant partial-onset seizures	USA	Drug Resistant Partial Onset Seizure. Age: 18 years and older. Sex: males and females	Terminated. 2015–2017	NCT02519439
**2**	Phase 3 Study of Adjunctive Ganaxolone in Adults With Drug-resistant Partial Onset Seizures and Open-label Extension	To determine the efficacy and safety of ganaxolone as adjunctive therapy for adults with drug-resistant partial-onset seizures followed by long-term open-label treatment	USA	Drug Resistant Partial Onset Seizure. Age: 18 years and older. Sex: males and females	Completed. 2015–2017	NCT01963208
**3**	A Randomized, Controlled Trial of Ganaxolone in Adult Uncontrolled Partial-Onset Seizures	To evaluate the effectiveness and safety of ganaxolone on partial seizure frequency in adults with epilepsy taking a maximum of 3 antiepileptic medications. The study will also evaluate the effectiveness of ganaxolone in females with catamenial epilepsy	USA	Partial Epilepsy; Catamenial Epilepsy. Age: 18–69 years. Sex: males and females	Completed. 2007–2009	NCT00465517
**4**	A Treatment Use Protocol for Subjects Continuing on From the Open-label Extension 0601 (0602)	To provide ganaxolone to those patients deriving significant benefit from current treatment in protocol 1042-0601	USA	Epilepsy, Complex Partial. Age: 18–55 years. Sex: males and females	Completed. 2009–2013	NCT01002820
**5**	A Multicenter, Open-Label Proof-of-Concept Trial of Ganaxolone in Children With PCDH19 Female Pediatric Epilepsy	To evaluate ganaxolone as adjunctive therapy for uncontrolled seizures in female children with PCDH19 mutations. After establishing baseline seizure frequency, qualifying subjects will enter the study and be treated with open-label ganaxolone for up to 6 months	USA	Epilepsy. Age: 2–10 years. Sex: females.	Currently recruiting. 2015	NCT02358538
**6**	Open-label Extension to Protocol 1042-0600	To evaluate efficacy and safety of ganaxolone treatment in adults with partial onset epilepsy with or without secondary generalizations	USA	Epilepsies, Partial. Age: 18–69 years. Sex: males and females	Completed. 2007–2013	NCT00512317
**7**	A Randomized, Controlled Trial of Ganaxolone in Patients With Infantile Spasms	To evaluate the safety, tolerability, and antiepileptic activity of ganaxolone in treatment of patients with infantile spasms	USA	Infantile Spasms. Age: 4–24 months. Sex: males and females	Completed. 2007–2009	NCT00441896

*Information obtained from ClinicalTrials.gov. May 2017*.

It is important to note that the histopathology of the hypoxic brain with a history of epilepsy varies from signs of gliosis and neuronal cell loss to impaired cellular maturation processes, some of which is regulated by microRNAs. In this review, we have not discussed the role of microRNAs in epilepsy. However, Dogini et al. (2013) and Jimenez-Mateos and Henshall (2013) have recently published an extensive review on the role of microRNAs in epilepsy, where the expression of microRNAs is largely dysregulated during neurogenesis, which is a major reason for drug resistance in epilepsy. Next generation sequencing has been instrumental in identifying pediatric patients with early onset severe epilepsy and any associated progressive brain injury (Mei et al., 2017). Long-term exposure of adult cultured cells to ganaxolone does not alter GABA^A^ receptor sensitivity, although it does alter GABA^A^ receptor composition by reducing the mRNA of α1 and γ2 subunits, and increasing α4 subunit mRNAs (Mascia et al., 2002). The consequences of these alterations of receptor subunit composition have not been investigated, but are likely to be significant. Further expression-profiling studies may reveal changes to brain miRNA levels following ganaxolone treatment in appropriate animal models of preterm or term seizure-induced brain injury, which will be of value to clinical studies.

## The GABA^A^ receptor switch

The GABAergic signaling is unique in that its action is dependent on the intracellular concentrations of chloride [Cl^−^], which can lead to either depolarization and excitation or hyperpolarization, and inhibition. The important regulators of Cl^−^ transport are NKCC1 and NKCC2 that respectively import and export Cl^−^. In adults, the interaction of neurosteroids such as ganaxolone with GABA^A^ receptors normally increases conductance of the chloride channel causing hyperpolarization, and inhibition of excitability. However, the role of GABA^A^ receptors in the regulation of excitability is dependent on neuronal maturation, synaptic plasticity, and pathological conditions, and there are notable species differences. For example, in rodent, during embryonic/fetal life and until at least postnatal life 9, immature neurons express the Na^+^ KCC1 (NKCC1) transporter, correlating with high internal [Cl^−^] in these neurons. On maturation, there is a decrease in NKCC1 transporter expression and an increase in expression of the K^+^/Cl^−^ (KCC2) transporter. These developmental changes coincide with a decrease in [Cl^−^], and GABA^A^ receptor stimulation then tends to hyperpolarize the cell causing inhibition (Delpire, 2000). In human neonates (and in other precocial species), greater presence of mature neurons leads to the GABA^A^ receptor becoming inhibitory, which is evident from at least 24–26 weeks (Ben-Ari et al., 2012), and in the latter part of gestation in other long gestation species. This indicates that the very premature infant may not be an ideal candidate for neurosteroid therapy, and it is therefore important to determine regulation of neuronal Cl^−^ in the very premature infant, and if the switch in GABA^A^ receptor function can be pharmacologically manipulated.

## The challenge

There is an opportunity to shift the paradigm of neonatal seizure management in changing both the timing of treatment and the agent used. Despite the acknowledged adverse side effects associated with phenobarbitone, it is still considered the initial drug of choice for the treatment of neonatal seizure, simply because options are limited. And because phenobarbitone-like drugs are known to have significant side effects, the reasonable caution in using them leads to delay in the treatment of neonatal seizures. We propose there are physiological reasons that synthetic pregnane steroids such as ganaxolone may be useful in suppressing neonatal seizures following hypoxic injury. Our studies with a relevant pre-clinical model (late gestation fetal sheep) have shown that endogenous neurosteroids that act through the GABA^A^ receptor pathway are neuroprotective, and they also function as effective anti-seizure agents. Therefore, further research into the anti-seizure properties of allopregnanolone analog with a GABA^A^ receptor agonist actions is warranted, particularly if it can be shown they avoid the side effects and reduced efficacy associated with the current use of phenobarbitone. The capacity to provide seizure control and neuroprotection with a safe agent administered early following hypoxia will improve the outcomes for our sickest neonates.

## Author contributions

TY, MF, and DW developed the idea. All authors collectively contributed to writing and drafting this mini review.

### Conflict of interest statement

The authors declare that the research was conducted in the absence of any commercial or financial relationships that could be construed as a potential conflict of interest.
